# Establishment and validation of a nomogram model for predicting early death in patients with endometrial cancer bone metastases

**DOI:** 10.3389/fonc.2025.1613843

**Published:** 2025-10-01

**Authors:** Qi Tang, Yating Sun, Yingchun Gao

**Affiliations:** Department of Gynecology, The Affiliated Huaian No. 1 People’s Hospital of Nanjing Medical University, Huai’an, China

**Keywords:** endometrial carcinoma, bone metastases, early death, SEER database, nomogram

## Abstract

**Background:**

Patients with endometrial cancer bone metastases (ECBM) are clinically rare and have a poor prognosis, including a higher incidence of early death (survival ≤ 3 months). Currently, no practical tools exist to predict early mortality in these patients. Thus, there is an urgent need to develop clinically applicable predictive models, such as nomograms, for individualized assessment of early death risk in ECBM.

**Methods:**

Relevant clinical and pathological data for ECBM patients from the SEER database (2010-2021). Univariate and multivariate logistic regression analyses were performed to identify risk factors associated with early death in ECBM patients and to construct prognostic nomograms. ROC analysis, calibration curves, and decision curve analysis (DCA) were used to assess the predictive accuracy and clinical utility of the nomogram model.

**Results:**

A total of 1,201 ECBM patients were found in the SEER database. After applying strict exclusion criteria, 769 patients were finally included in this study. Patients were randomly divided into training and validation cohorts in a 7:3 ratio. The results of univariate and multivariate logistic regression analyses revealed several independent predictive factors for early death. For both overall early death (OED) and cancer-specific early death (CSED), protective factors included surgery (OED: OR = 0.22, 95%CI: 0.12-0.41, p<0.001; CSED: OR = 0.33, 95%CI: 0.18-0.61, p<0.001) and chemotherapy (OED: OR = 0.11, 95%CI: 0.06-0.18, p<0.001; CSED: OR = 0.14, 95%CI: 0.09-0.24, p<0.001). Brain metastases increased risk (OED: OR = 2.98, 95%CI: 1.29-6.87, p=0.01; CSED: OR = 2.20, 95%CI: 1.04-4.79, p=0.047). Compared to 0–9 days, longer time from diagnosis to treatment showed protective associations: 10–27 days (OED: OR = 0.51, 95%CI: 0.27-0.98, p=0.042) and ≥28 days (OED: OR = 0.23, 95%CI: 0.12-0.44, p<0.001; CSED: OR = 0.30, 95%CI: 0.16-0.56, p<0.001). Regarding histological type, compared to endometrioid subtype, sarcomatous subtype significantly increased OED risk (OR = 3.04, 95%CI: 1.40-6.57, p=0.005), while radiotherapy reduced CSED risk (OR = 0.55, 95%CI: 0.33-0.92, p=0.022). Based on these variables, nomograms were developed to predict the risk of early death. The ROC curve confirmed the model’s high predictive accuracy, while the calibration curve showed strong alignment between predicted and actual survival. DCA further demonstrated its clinical utility.

**Conclusion:**

In this study, we developed robust nomogram models to predict the probability of early death in ECBM patients.

## 1 Introduction

Endometrial cancer (EC) is one of the most common malignant tumors of the female reproductive system, with an increasing incidence and mortality rate each year. According to the American Cancer Society, in 2022, the number of new cases in the United States rose to 65,950, resulting in 12,550 deaths and posing a significant threat to women’s health (1, 2). Although most EC patients are diagnosed early and have a favorable prognosis, with a 5-year overall survival rate of approximately 80%, those advanced or recurrent patients have a poor prognosis. Distant metastasis is the primary cause of death in EC patients, with a 5-year survival rate of less than 20% (3, 4). Bone and brain are the least common distant metastases organs for EC patients, with a median survival time of only 4 months for ECBM patients (5).

Currently, the TNM staging system established by the American Joint Committee on Cancer (AJCC) is the standard tool for predicting survival rates in EC patients. However, its predictive effectiveness significantly diminishes when applied to metastatic disease (6). Some ECBM patients die within 3 months of diagnosis. To date, there have been relatively few reports on ECBM, and no prognostic models specifically designed to predict early death have been established. Recent studies have indicated that nomograms offer a convenient and accurate tool for assessing the prognosis of cancer (7).

This study utilizes a large sample of clinical data from the Surveillance, Epidemiology, and End Results (SEER) database of the National Cancer Institute to analyze clinical and pathological factors associated with prognosis in ECBM patients. Nomogram models were constructed to predict the risk of early death (8). This model is designed to enhance the assessment of prognosis in ECBM patients, enabling clinicians to identify high-risk individuals timely, and develop personalized treatment plans, ultimately aiming to extend life expectancy and improve patients’ quality of life.

## 2 Methods

Data from 769 ECBM patients were extracted from the SEER database (URL: https://seer.cancer.gov/) covering the period from 2010 to 2021. The study extracted basic clinical and pathological information as well as treatment methods for the patients. Authorization for access to and use of the SEER database has been obtained for this research. Given the anonymized nature and publicly accessible data within the SEER database, this study does not require additional approval from an institutional ethics committee. This research adheres to the ethical standards outlined in the 1964 Declaration of Helsinki and its subsequent amendments and similar ethical guidelines.

### 2.1 Patient cohorts

**Figure 1:**
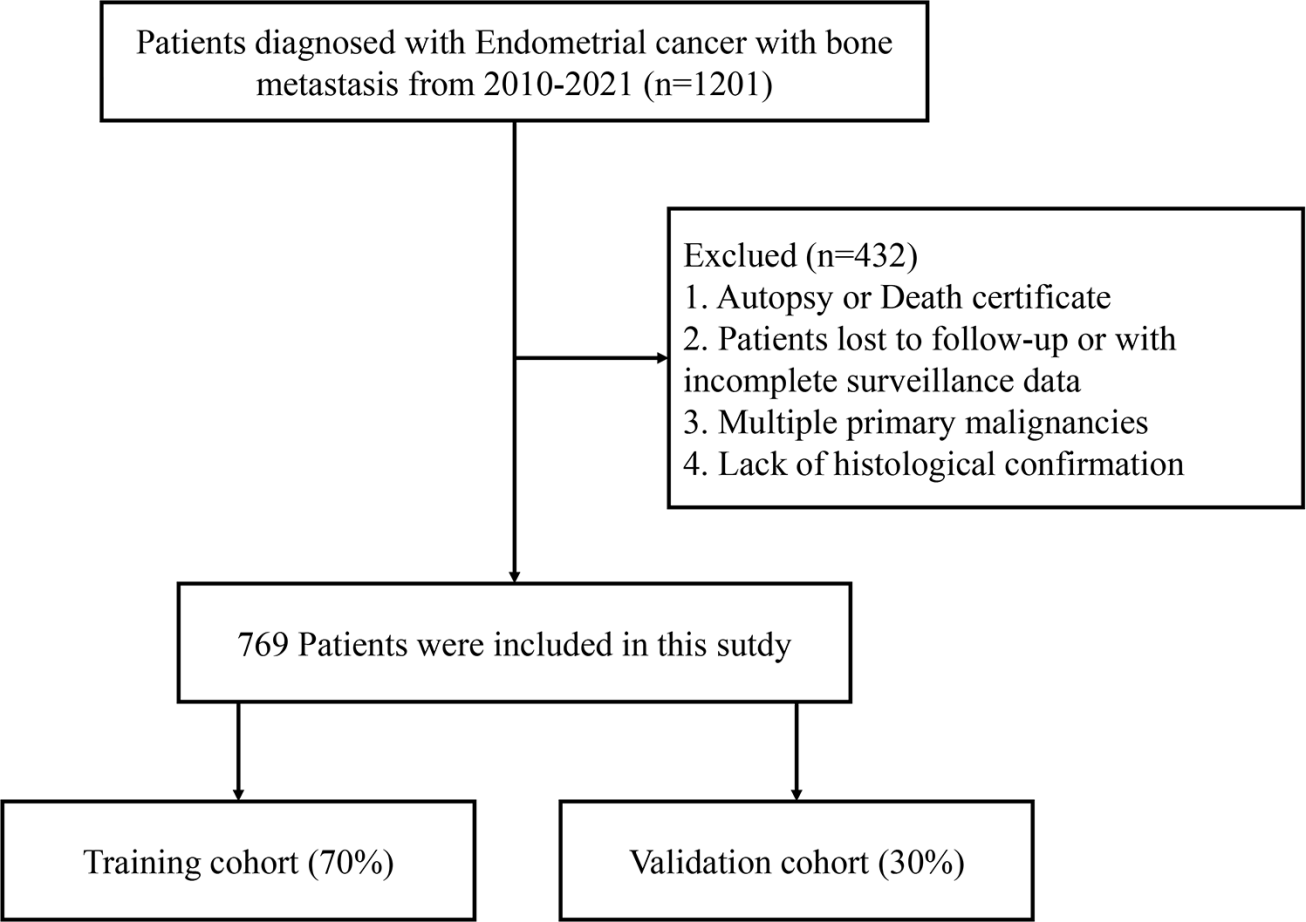
The flow chart of ECBM patient enrollment.

The clinical information of 769 patients was extracted by the SEER*Stat database (version 8.4.4). The inclusion criteria were as follows: (1) pathological diagnosis of endometrial cancer; (2) with bone metastasis; (3) only primary cancer; (4) diagnosis from 2010 to 2021. The exclusion criteria were as follows: (1) patients with unknown survival time; (2) patients with unspecified histological type; (3) patients with multiple primary tumors; and (4) patients with missing racial information. The selection process of the ECBM patients is shown in **Figure 1**.

### 2.2 Data collection

The exclusion studies follow the criteria: (a) study children with cancer and cancer patients undergoing treatment; (b) intervention studies combining exercise with cognitive therapy, physiotherapy, massaging, diet, or medication; (c) studies that excluded trials with no control group; (d) Endpoints that reported cancer-specific scales and excluded non-cancer survivors’ HRQoL scales. The demographic information includes age, race, marital status, tumor size, histological grade, histological type, TNM staging, metastases, the time from diagnosis of ECBM to treatment, the year of diagnosis, surgery, radiotherapy, and chemotherapy.

According to the International Classification of Diseases for Oncology, Third Edition (ICD-O-3), metastatic EC patients were divided into three histological subtypes: endometrioid (codes 8380, 8382), non-endometrioid (codes 8000, 8010, 8013, 8020, 8041, 8045, 8046, 8050, 8070, 8140, 8246, 8255, 8260, 8263, 8310, 8323, 8441, 8460, 8461, 8480, 8560, 8570, 8574), and sarcomatous (codes 8800, 8802, 8805, 8890, 8900, 8902, 8930, 8933, 8935, 8950, 8980, 9102). Tumor grading was defined as Grade I (well-differentiated), Grade II (moderately differentiated), Grade III (poorly differentiated), and Grade IV (undifferentiated).

Based on previous studies, OED was defined as death from any cause occurring within three months (9). CSED refers to death resulting from ECBM within the same three-month period. The study endpoints were OED and CSED. Survival time was calculated from the date of initial diagnosis of ECBM.

### 2.3 Statistical analyses

The eligible patients were randomly divided into a training cohort (537 patients) and a validation cohort (232 patients) in a 7:3 ratio. Statistical analyses were conducted by using SPSS 27.0 and R 4.4.1 software. Categorical variables were presented as frequencies (percentages), and intergroup comparisons were performed using the chi-square test or Fisher’s exact test. Ordered categorical variables were compared using the rank-sum test. The X-tile software was utilized to determine the optimal cutoff values for age, tumor size, and time from diagnosis to treatment for ECBM patients. Univariate and multivariate logistic regression analyses were performed to identify independent risk factors for OED and CSED within the training cohort. Nomograms were constructed to predict OED and CSED. The predictive models’ discrimination and diagnostic performance were assessed using ROC curves and AUC values. The calibration and clinical applicability of the models were evaluated through calibration curves and DCA analysis. A P-value of <0.05 was considered statistically significant.

## 3 Results

### 3.1 Determine the age, tumor size, and optimal cutoff from diagnosis to treatment based on X-tile

**Figure 2:**
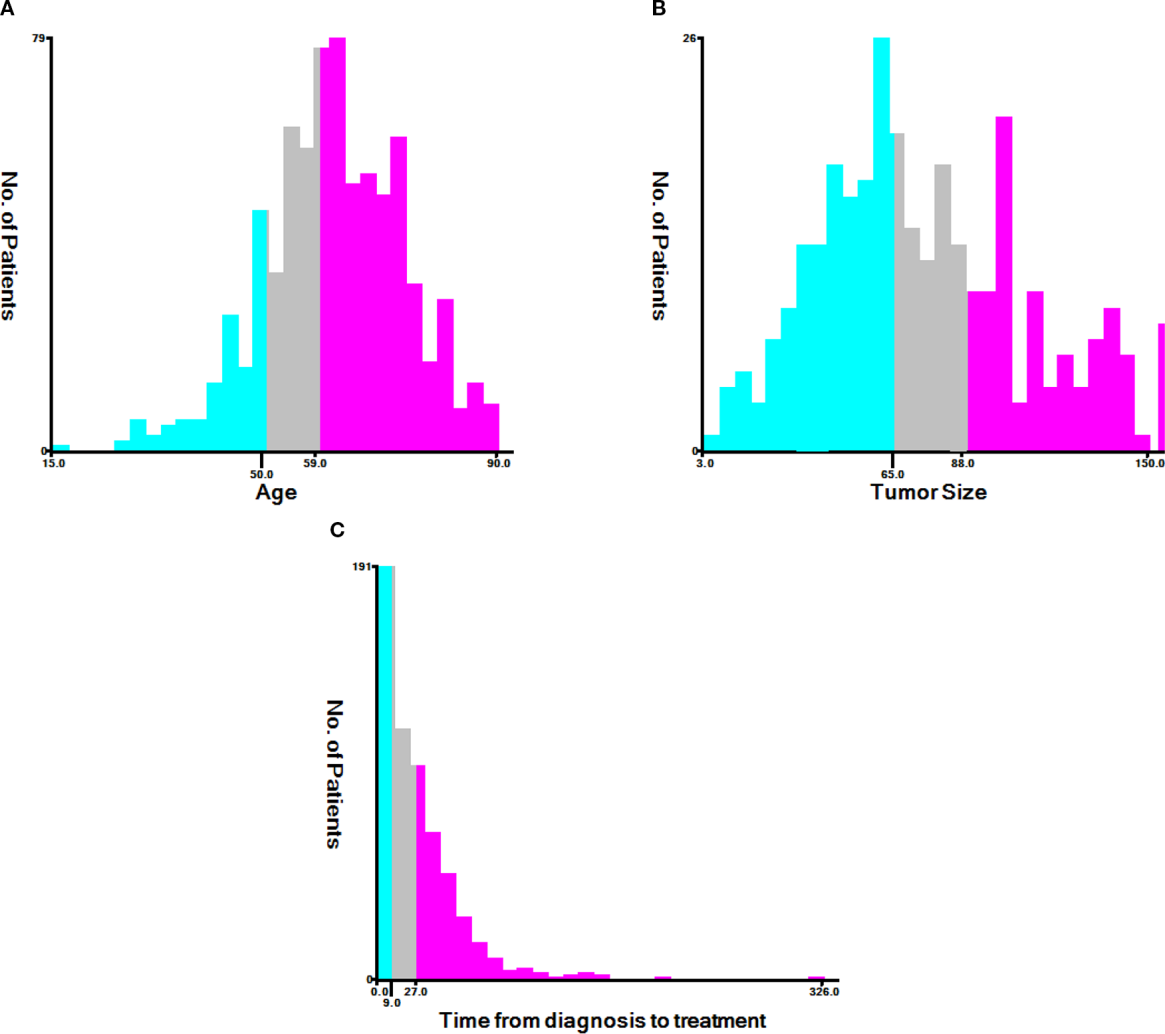
The X-tile analysis. **(A)** Age, **(B)** tumor size, **(C)** time from diagnosis to treatment.

According to X-tile, the optimal cutoff values for age were 50 years old and 59 years old. Patients were categorized into groups of ≤50, 51–59, and ≥60 years old (**Figure 2A**). The optimal cutoff values for tumor size were identified as 65 mm and 88 mm, with patients grouped as ≤ 65 mm, 66–88 mm, and ≥ 89 mm (**Figure 2B**). Additionally, the optimal cutoff values for time from diagnosis to treatment were established at 9 days and 27 days, resulting in patient groupings of ≤9 days, 10–27 days, and ≥28 days (**Figure 2C**).

### 3.2 Epidemiological and clinicopathological features

According to the inclusion and exclusion criteria, a total of 769 ECBM patients were retrospectively included in this study. As shown in **Table 1**, 41.5% (319/769) of the ECBM patients died within three months, with 38.9% (299/769) dying from ECBM, and most of the patients were White (80.6%). Lung metastases (55.5%) were more common than brain metastases (10.0%) and liver metastases (31.7%). The most common histological type was non-endometrioid (58.0%), followed by the endometrioid (27.0%) and sarcomatous (15.0%). Additionally, 32.0% of patients received radiotherapy, 23.8% underwent chemotherapy, while only 17.2% received surgical treatment. The patients were randomly divided into a training cohort (*n* = 537) and an internal validation cohort (*n* = 232) in a 7:3 ratio. Comparisons of demographic and clinicopathological parameters between the training and validation cohorts showed no statistically significant differences (P > 0.05), indicating that the cohorts were suitable for subsequent analyses (**Table 2**).

**Table 1:** Epidemiological and clinicopathological characteristics of ECBM patients.

Name	Levels	No (N=450)	Overall early death (N=319)	Cancer-specific early death (N=299)
Age	15-50	70 (15.6%)	36 (11.3%)	35 (11.7%)
	51-59	116 (25.8%)	81 (25.4%)	73 (24.4%)
	>=60	264 (58.7%)	202 (63.3%)	191 (63.9%)
Race	White	361 (80.2%)	257 (80.6%)	243 (81.3%)
	Black	42 (9.3%)	23 (7.2%)	22 (7.4%)
	Other	47 (10.4%)	39 (12.2%)	34 (11.4%)
Marital.status	Married	218 (48.4%)	157 (49.2%)	148 (49.5%)
	Unmarried	216 (48%)	148 (46.4%)	138 (46.2%)
	unknown	16 (3.6%)	14 (4.4%)	13 (4.3%)
Grade	I	9 (2%)	4 (1.3%)	4 (1.3%)
	II	43 (9.6%)	12 (3.8%)	12 (4%)
	III	137 (30.4%)	78 (24.5%)	71 (23.7%)
	IV	65 (14.4%)	41 (12.9%)	39 (13%)
	unknown	196 (43.6%)	184 (57.7%)	173 (57.9%)
T	T1	49 (10.9%)	27 (8.5%)	25 (8.4%)
	T2	41 (9.1%)	19 (6%)	19 (6.4%)
	T3	193 (42.9%)	90 (28.2%)	80 (26.8%)
	T4	40 (8.9%)	41 (12.9%)	38 (12.7%)
	unknown	127 (28.2%)	142 (44.5%)	137 (45.8%)
N	N0	128 (28.4%)	89 (27.9%)	80 (26.8%)
	N1	152 (33.8%)	106 (33.2%)	100 (33.4%)
	N3	92 (20.4%)	53 (16.6%)	50 (16.7%)
	unknown	78 (17.3%)	71 (22.3%)	69 (23.1%)
Surgery	No	271 (60.2%)	264 (82.8%)	248 (82.9%)
	Yes	179 (39.8%)	55 (17.2%)	51 (17.1%)
Radiation	No/Unknown	219 (48.7%)	217 (68%)	205 (68.6%)
	Yes	231 (51.3%)	102 (32%)	94 (31.4%)
Chemotherapy	No/Unknown	106 (23.6%)	243 (76.2%)	228 (76.3%)
	Yes	344 (76.4%)	76 (23.8%)	71 (23.7%)
Tumor.Size	3-65	101 (22.4%)	43 (13.5%)	40 (13.4%)
	66-88	42 (9.3%)	23 (7.2%)	21 (7%)
	90-150	56 (12.4%)	43 (13.5%)	40 (13.4%)
	unknown	251 (55.8%)	210 (65.8%)	198 (66.2%)
Brain.metastasis	No	417 (92.7%)	277 (86.8%)	261 (87.3%)
	Yes	23 (5.1%)	32 (10%)	29 (9.7%)
	Unknown	10 (2.2%)	10 (3.1%)	9 (3%)
Liver.metastasis	No	330 (73.3%)	209 (65.5%)	196 (65.6%)
	Yes	117 (26%)	101 (31.7%)	95 (31.8%)
	Unknown	3 (0.7%)	9 (2.8%)	8 (2.7%)
Lung.metastasis	No	232 (51.6%)	132 (41.4%)	121 (40.5%)
	Yes	207 (46%)	177 (55.5%)	168 (56.2%)
	Unknown	11 (2.4%)	10 (3.1%)	10 (3.3%)
Time.from.diagnosis.to.treatment	0-9	88 (19.6%)	75 (23.5%)	69 (23.1%)
	10-27	124 (27.6%)	62 (19.4%)	59 (19.7%)
	28-326	201 (44.7%)	51 (16%)	47 (15.7%)
	unknown	37 (8.2%)	131 (41.1%)	124 (41.5%)
Year.of.diagnosis	2010-2015	201 (44.7%)	134 (42%)	124 (41.5%)
	2016-2021	249 (55.3%)	185 (58%)	175 (58.5%)
Histology	endometrioid subtype	156 (34.7%)	86 (27%)	79 (26.4%)
	non-endometrioid subtype	236 (52.4%)	185 (58%)	177 (59.2%)
	sarcoma subtype	58 (12.9%)	48 (15%)	43 (14.4%)

(Grade: I (highly differentiated), II (moderately differentiated), III/IV (poorly differentiated or undifferentiated)).

**Table 2:** The comparison of characteristics of ECBM patients in the training and validation cohorts.

Name	Levels	Training (N=537)	Validation (N=232)	p
Age	15-50	77 (14.3%)	29 (12.5%)	.793
	51-59	137 (25.5%)	60 (25.9%)	
	>=60	323 (60.1%)	143 (61.6%)	
Race	White	424 (79%)	194 (83.6%)	.209
	Black	46 (8.6%)	19 (8.2%)	
	Other	67 (12.5%)	19 (8.2%)	
Marital.status	Married	266 (49.5%)	109 (47%)	.644
	Unmarried	252 (46.9%)	112 (48.3%)	
	unknown	19 (3.5%)	11 (4.7%)	
Grade	I	11 (2%)	2 (0.9%)	.649
	II	38 (7.1%)	17 (7.3%)	
	III	146 (27.2%)	69 (29.7%)	
	IV	78 (14.5%)	28 (12.1%)	
	unknown	264 (49.2%)	116 (50%)	
T	T1	49 (9.1%)	27 (11.6%)	.136
	T2	43 (8%)	17 (7.3%)	
	T3	186 (34.6%)	97 (41.8%)	
	T4	63 (11.7%)	18 (7.8%)	
	unknown	196 (36.5%)	73 (31.5%)	
N	N0	156 (29.1%)	61 (26.3%)	.172
	N1	176 (32.8%)	82 (35.3%)	
	N3	93 (17.3%)	52 (22.4%)	
	unknown	112 (20.9%)	37 (15.9%)	
Surgery	No	380 (70.8%)	155 (66.8%)	.313
	Yes	157 (29.2%)	77 (33.2%)	
Radiation	No/Unknown	312 (58.1%)	124 (53.4%)	.265
	Yes	225 (41.9%)	108 (46.6%)	
Chemotherapy	No/Unknown	244 (45.4%)	105 (45.3%)	1.000
	Yes	293 (54.6%)	127 (54.7%)	
Tumor.Size	3-65	102 (19%)	42 (18.1%)	.507
	66-88	41 (7.6%)	24 (10.3%)	
	90-150	66 (12.3%)	33 (14.2%)	
	unknown	328 (61.1%)	133 (57.3%)	
Brain.metastasis	No	483 (89.9%)	211 (90.9%)	.359
	Yes	42 (7.8%)	13 (5.6%)	
	Unknown	12 (2.2%)	8 (3.4%)	
Liver.metastasis	No	371 (69.1%)	168 (72.4%)	.595
	Yes	158 (29.4%)	60 (25.9%)	
	Unknown	8 (1.5%)	4 (1.7%)	
Lung.metastasis	No	245 (45.6%)	119 (51.3%)	.301
	Yes	278 (51.8%)	106 (45.7%)	
	Unknown	14 (2.6%)	7 (3%)	
Time.from.diagnosis.to.treatment	0-9	116 (21.6%)	47 (20.3%)	.447
	10-27	121 (22.5%)	65 (28%)	
	28-326	180 (33.5%)	72 (31%)	
	unknown	120 (22.3%)	48 (20.7%)	
Year.of.diagnosis	2010-2015	237 (44.1%)	98 (42.2%)	.684
	2016-2021	300 (55.9%)	134 (57.8%)	
Histology	endometrioid subtype	171 (31.8%)	71 (30.6%)	.934
	non-endometrioid subtype	293 (54.6%)	128 (55.2%)	
	sarcoma subtype	73 (13.6%)	33 (14.2%)	

### 3.3 Univariate and multivariate logistic regression analysis

Univariate analysis revealed that surgery, chemotherapy, radiotherapy, brain metastasis, lung metastasis, liver metastasis, time from diagnosis to treatment, and histological type were significantly associated with OED (all p<0.05). Similarly, surgery, chemotherapy, radiotherapy, brain metastasis, lung metastasis, liver metastasis, and time from diagnosis to treatment were significantly associated with CSED (all p<0.05).

Multivariate logistic regression analysis identified independent predictive factors for early death. For OED, protective factors included surgery (OR = 0.22, 95%CI: 0.12-0.41, p<0.001) and chemotherapy (OR = 0.11, 95%CI: 0.06-0.18, p<0.001). Brain metastasis was associated with increased risk (OR = 2.98, 95%CI: 1.29-6.87, p=0.01). Interestingly, compared to patients treated within 9 days, longer time from diagnosis to treatment was associated with reduced risk: 10–27 days (OR = 0.51, 95%CI: 0.27-0.98, p=0.042) and ≥28 days (OR = 0.23, 95%CI: 0.12-0.44, p<0.001). Regarding histological type, using endometrioid as reference, the sarcomatous subtype significantly increased OED risk (OR = 3.04, 95%CI: 1.40-6.57, p=0.005), while non-endometrioid subtype showed no significant difference.

For CSED, protective factors included surgery (OR = 0.33, 95%CI: 0.18-0.61, p<0.001), chemotherapy (OR = 0.14, 95%CI: 0.09-0.24, p<0.001), and radiotherapy (OR = 0.55, 95%CI: 0.33-0.92, p=0.022). Brain metastasis increased risk (OR = 2.20, 95%CI: 1.04-4.79, p=0.047). Similar to OED, longer time from diagnosis to treatment (≥28 days) was associated with reduced CSED risk (OR = 0.30, 95%CI: 0.16-0.56, p<0.001). The paradoxical protective effect of longer time to treatment may reflect immortal time bias or selection of patients with better performance status who could afford treatment delays (**Tables 3**, **4**).

**Table 3:** Univariate and multivariate Logistic regression analysis of OED in the ECBM patients.

Dependent: overall early death		0 (N=309)	1 (N=228)	OR (95%CI) (univariable)	OR (95%CI) (multivariable)
Age	15-50	46 (14.9%)	31 (13.6%)		
	51-59	86 (27.8%)	51 (22.4%)	0.88 (0.50-1.56, p=.661)	
	>=60	177 (57.3%)	146 (64%)	1.22 (0.74-2.03, p=.433)	
Race	White	242 (78.3%)	182 (79.8%)		
	Black	30 (9.7%)	16 (7%)	0.71 (0.38-1.34, p=.290)	
	Other	37 (12%)	30 (13.2%)	1.08 (0.64-1.81, p=.776)	
Marital.status	Married	156 (50.5%)	110 (48.2%)		
	Unmarried	144 (46.6%)	108 (47.4%)	1.06 (0.75-1.51, p=.729)	
	unknown	9 (2.9%)	10 (4.4%)	1.58 (0.62-4.01, p=.340)	
Grade	I	8 (2.6%)	3 (1.3%)		
	II	30 (9.7%)	8 (3.5%)	0.71 (0.15-3.31, p=.664)	
	III	95 (30.7%)	51 (22.4%)	1.43 (0.36-5.63, p=.608)	
	IV	48 (15.5%)	30 (13.2%)	1.67 (0.41-6.78, p=.475)	
	unknown	128 (41.4%)	136 (59.6%)	2.83 (0.74-10.91, p=.130)	
T	T1	30 (9.7%)	19 (8.3%)		
	T2	30 (9.7%)	13 (5.7%)	0.68 (0.29-1.63, p=.392)	0.63 (0.21-1.90, p=.415)
	T3	126 (40.8%)	60 (26.3%)	0.75 (0.39-1.44, p=.391)	0.60 (0.26-1.39, p=.233)
	T4	34 (11%)	29 (12.7%)	1.35 (0.63-2.88, p=.442)	0.99 (0.37-2.64, p=.979)
	unknown	89 (28.8%)	107 (46.9%)	1.90 (1.00-3.60, p=.050)	0.66 (0.28-1.53, p=.330)
N	N0	93 (30.1%)	63 (27.6%)		
	N1	101 (32.7%)	75 (32.9%)	1.10 (0.71-1.70, p=.681)	
	N3	59 (19.1%)	34 (14.9%)	0.85 (0.50-1.44, p=.549)	
	unknown	56 (18.1%)	56 (24.6%)	1.48 (0.90-2.41, p=.119)	
Surgery	No	189 (61.2%)	191 (83.8%)		
	Yes	120 (38.8%)	37 (16.2%)	0.31 (0.20-0.46, p<.001)	0.22 (0.12-0.41, p<.001)
Radiation	No/Unknown	161 (52.1%)	151 (66.2%)		
	Yes	148 (47.9%)	77 (33.8%)	0.55 (0.39-0.79, p=.001)	0.59 (0.35-1.00, p=.051)
Chemotherapy	No/Unknown	72 (23.3%)	172 (75.4%)		
	Yes	237 (76.7%)	56 (24.6%)	0.10 (0.07-0.15, p<.001)	0.11 (0.06-0.18, p<.001)
Tumor.Size	3-65	70 (22.7%)	32 (14%)		
	66-88	26 (8.4%)	15 (6.6%)	1.26 (0.59-2.70, p=.549)	1.81 (0.65-5.04, p=.258)
	90-150	39 (12.6%)	27 (11.8%)	1.51 (0.79-2.89, p=.207)	1.21 (0.53-2.78, p=.648)
	unknown	174 (56.3%)	154 (67.5%)	1.94 (1.21-3.10, p=.006)	1.04 (0.56-1.93, p=.905)
Brain.metastasis	No	286 (92.6%)	197 (86.4%)		
	Yes	17 (5.5%)	25 (11%)	2.13 (1.12-4.06, p=.021)	2.98 (1.29-6.87, p=.010)
	Unknown	6 (1.9%)	6 (2.6%)	1.45 (0.46-4.57, p=.524)	0.21 (0.01-3.60, p=.281)
Liver.metastasis	No	227 (73.5%)	144 (63.2%)		
	Yes	81 (26.2%)	77 (33.8%)	1.50 (1.03-2.18, p=.035)	1.59 (0.96-2.63, p=.071)
	Unknown	1 (0.3%)	7 (3.1%)	11.03 (1.34-90.62, p=.025)	23.07 (0.55-962.35, p=.099)
Lung.metastasis	No	154 (49.8%)	91 (39.9%)		
	Yes	148 (47.9%)	130 (57%)	1.49 (1.05-2.11, p=.026)	1.17 (0.74-1.85, p=.504)
	Unknown	7 (2.3%)	7 (3.1%)	1.69 (0.58-4.98, p=.339)	0.58 (0.10-3.19, p=.527)
Time.from.diagnosis.to.treatment	0-9	58 (18.8%)	58 (25.4%)		
	10-27	82 (26.5%)	39 (17.1%)	0.48 (0.28-0.81, p=.006)	0.51 (0.27-0.98, p=.042)
	28-326	142 (46%)	38 (16.7%)	0.27 (0.16-0.45, p<.001)	0.23 (0.12-0.44, p<.001)
	unknown	27 (8.7%)	93 (40.8%)	3.44 (1.96-6.04, p<.001)	0.69 (0.30-1.55, p=.366)
Year.of.diagnosis	2010-2015	142 (46%)	95 (41.7%)		
	2016-2021	167 (54%)	133 (58.3%)	1.19 (0.84-1.68, p=.323)	
Histology	endometrioid subtype	110 (35.6%)	61 (26.8%)		
	non-endometrioid subtype	163 (52.8%)	130 (57%)	1.44 (0.98-2.12, p=.067)	1.42 (0.85-2.37, p=.184)
	sarcoma subtype	36 (11.7%)	37 (16.2%)	1.85 (1.06-3.23, p=.029)	3.04 (1.40-6.57, p=.005)

((0: survival > 3 months; 1: survival ≤3 months); (OR: Odds Ratio; 95%CI: 95% Confidence Interval)). OR <1 indicates protective association (reduced risk of early death); OR >1 indicates adverse association (increased risk of early death).

**Table 4:** Univariate and multivariate Logistic regression analysis of CSED in the ECBM patients.

Dependent: cancer-specific early death		0 (N=323)	1 (N=214)	OR (95%CI) (univariable)	OR (95%CI) (multivariable)
Age	15-50	47 (14.6%)	30 (14%)		
	51-59	92 (28.5%)	45 (21%)	0.77 (0.43-1.37, p=.369)	
	>=60	184 (57%)	139 (65%)	1.18 (0.71-1.97, p=.516)	
Race	White	251 (77.7%)	173 (80.8%)		
	Black	30 (9.3%)	16 (7.5%)	0.77 (0.41-1.46, p=.430)	
	Other	42 (13%)	25 (11.7%)	0.86 (0.51-1.47, p=.589)	
Marital.status	Married	162 (50.2%)	104 (48.6%)		
	Unmarried	152 (47.1%)	100 (46.7%)	1.02 (0.72-1.46, p=.892)	
	unknown	9 (2.8%)	10 (4.7%)	1.73 (0.68-4.40, p=.249)	
Grade	I	8 (2.5%)	3 (1.4%)		
	II	30 (9.3%)	8 (3.7%)	0.71 (0.15-3.31, p=.664)	
	III	100 (31%)	46 (21.5%)	1.23 (0.31-4.84, p=.770)	
	IV	49 (15.2%)	29 (13.6%)	1.58 (0.39-6.43, p=.524)	
	unknown	136 (42.1%)	128 (59.8%)	2.51 (0.65-9.67, p=.181)	
T	T1	32 (9.9%)	17 (7.9%)		
	T2	30 (9.3%)	13 (6.1%)	0.82 (0.34-1.96, p=.649)	0.84 (0.29-2.42, p=.746)
	T3	132 (40.9%)	54 (25.2%)	0.77 (0.39-1.50, p=.443)	0.64 (0.28-1.46, p=.283)
	T4	37 (11.5%)	26 (12.1%)	1.32 (0.61-2.87, p=.478)	0.93 (0.35-2.45, p=.876)
	unknown	92 (28.5%)	104 (48.6%)	2.13 (1.11-4.08, p=.023)	1.00 (0.43-2.32, p=.997)
N	N0	100 (31%)	56 (26.2%)		
	N1	105 (32.5%)	71 (33.2%)	1.21 (0.77-1.88, p=.406)	1.20 (0.68-2.13, p=.521)
	N3	60 (18.6%)	33 (15.4%)	0.98 (0.57-1.68, p=.948)	1.04 (0.53-2.06, p=.900)
	unknown	58 (18%)	54 (25.2%)	1.66 (1.01-2.73, p=.044)	0.81 (0.42-1.58, p=.538)
Surgery	No	201 (62.2%)	179 (83.6%)		
	Yes	122 (37.8%)	35 (16.4%)	0.32 (0.21-0.49, p<.001)	0.33 (0.18-0.61, p<.001)
Radiation	No/Unknown	168 (52%)	144 (67.3%)		
	Yes	155 (48%)	70 (32.7%)	0.53 (0.37-0.75, p<.001)	0.55 (0.33-0.92, p=.022)
Chemotherapy	No/Unknown	83 (25.7%)	161 (75.2%)		
	Yes	240 (74.3%)	53 (24.8%)	0.11 (0.08-0.17, p<.001)	0.14 (0.09-0.24, p<.001)
Tumor.Size	3-65	72 (22.3%)	30 (14%)		
	66-88	28 (8.7%)	13 (6.1%)	1.11 (0.51-2.44, p=.787)	1.43 (0.53-3.90, p=.483)
	90-150	41 (12.7%)	25 (11.7%)	1.46 (0.76-2.82, p=.254)	1.35 (0.60-3.03, p=.473)
	unknown	182 (56.3%)	146 (68.2%)	1.93 (1.19-3.11, p=.007)	1.04 (0.57-1.91, p=.902)
Brain.metastasis	No	298 (92.3%)	185 (86.4%)		
	Yes	19 (5.9%)	23 (10.7%)	1.95 (1.03-3.68, p=.039)	2.20 (1.01-4.79, p=.047)
	Unknown	6 (1.9%)	6 (2.8%)	1.61 (0.51-5.07, p=.415)	0.23 (0.02-3.26, p=.277)
Liver.metastasis	No	237 (73.4%)	134 (62.6%)		
	Yes	85 (26.3%)	73 (34.1%)	1.52 (1.04-2.22, p=.030)	1.60 (0.98-2.62, p=.059)
	Unknown	1 (0.3%)	7 (3.3%)	12.38 (1.51-101.71, p=.019)	26.12 (0.78-879.02, p=.069)
Lung.metastasis	No	161 (49.8%)	84 (39.3%)		
	Yes	155 (48%)	123 (57.5%)	1.52 (1.07-2.17, p=.020)	1.18 (0.76-1.84, p=.468)
	Unknown	7 (2.2%)	7 (3.3%)	1.92 (0.65-5.65, p=.238)	0.60 (0.11-3.39, p=.562)
Time.from.diagnosis.to.treatment	0-9	64 (19.8%)	52 (24.3%)		
	10-27	84 (26%)	37 (17.3%)	0.54 (0.32-0.92, p=.024)	0.63 (0.33-1.19, p=.153)
	28-326	144 (44.6%)	36 (16.8%)	0.31 (0.18-0.52, p<.001)	0.30 (0.16-0.56, p<.001)
	unknown	31 (9.6%)	89 (41.6%)	3.53 (2.04-6.12, p<.001)	0.83 (0.39-1.79, p=.637)
Year.of.diagnosis	2010-2015	151 (46.7%)	86 (40.2%)		
	2016-2021	172 (53.3%)	128 (59.8%)	1.31 (0.92-1.85, p=.134)	
Histology	endometrioid subtype	114 (35.3%)	57 (26.6%)		
	non-endometrioid subtype	169 (52.3%)	124 (57.9%)	1.47 (0.99-2.17, p=.056)	
	sarcoma subtype	40 (12.4%)	33 (15.4%)	1.65 (0.94-2.89, p=.080)	

((0: CSED; 1: non-CSED); (OR, Odds Ratio; 95%CI, 95% Confidence Interval)).

### 3.4 Establishment and verification of nomograms

**Figure 3:**
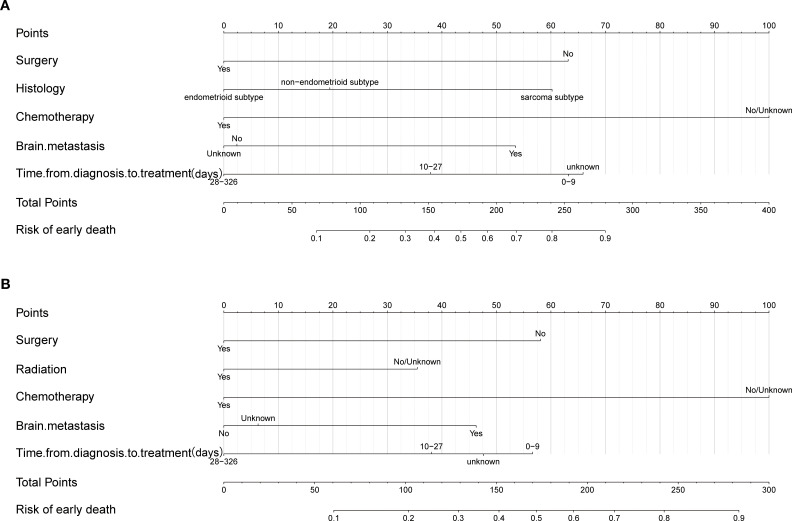
Nomogram for predicting early death in ECBM patients. **(A)** Overall early death, **(B)** cancer-specific early death.

**Figure 4:**
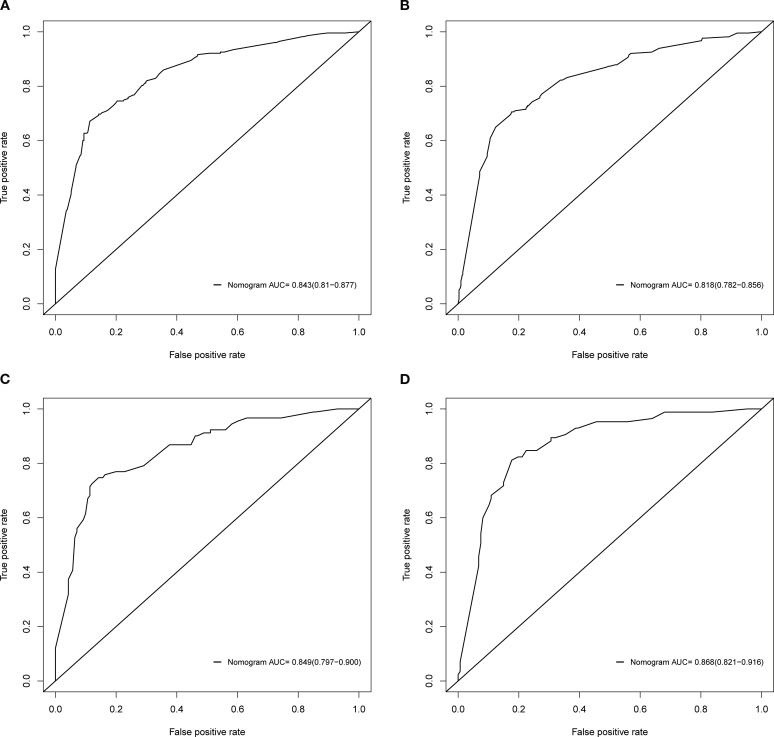
ROC analysis of the nomograms. **(A)** OED in the training cohort, **(B)** CSED in the training cohort, **(C)** OED in the validation cohort, **(D)** CSED in the validation cohort.

**Figure 5:**
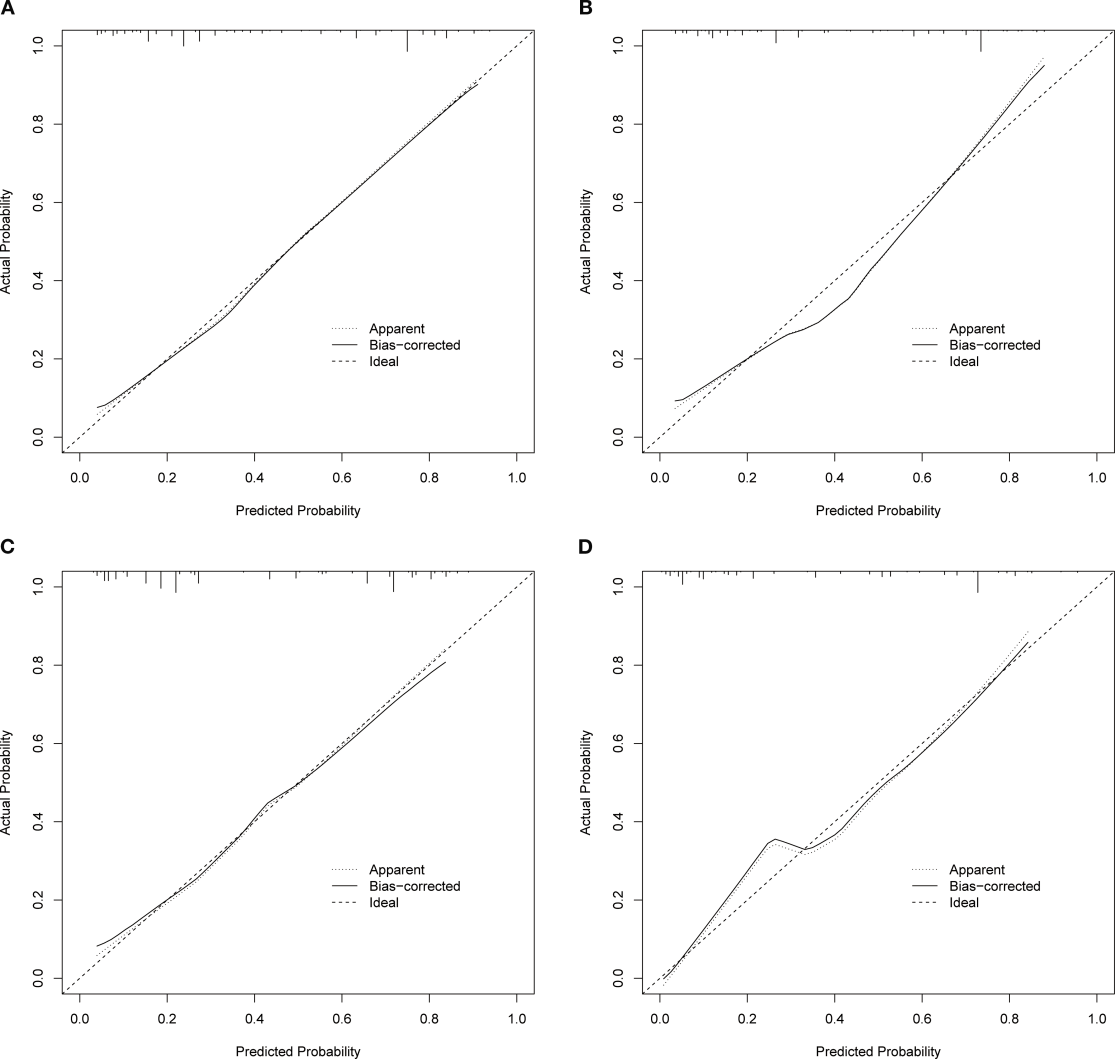
Calibration curves. **(A)** OED in the training cohort, **(B)** CSED in the training cohort, **(C)** OED in the validation cohort, **(D)** CSED in the validation cohort.

**Figure 6:**
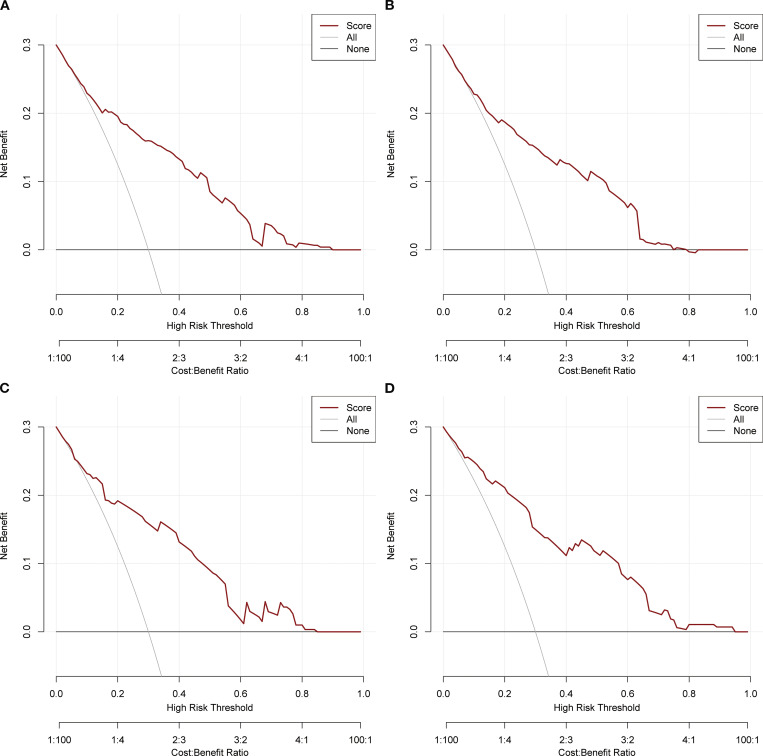
DCA analysis. **(A)** OED in the training cohort, **(B)** CSED in the training cohort, **(C)** OED in the validation cohort, **(D)** CSED in the validation cohort.

Based on the independent factors identified by univariate and multivariate logistic regression analysis. Nomograms were developed to evaluate the risks of OED and CSED in ECBM patients. (**Figure 3**). The ROC analysis for OED and CSED in the training and validation cohorts were shown in **Figure 4**. In the training cohort, the AUC values for OED and CSED were 0.843 and 0.818, respectively. In the validation cohort, the AUC values for OED and CSED were 0.849 and 0.868, respectively, indicating that the nomograms demonstrated strong predictive performance. The calibration curves demonstrate a strong concordance between predicted and observed probabilities (**Figure 5**). DCA analysis indicated that the model provides a positive net benefit, suggesting that the models developed in this study have substantial clinical applicability (**Figure 6**).

## 4 Discussion

This study revealed that the early death rate in ECBM patients is alarmingly high, reaching 41.5%. This finding underscores the urgent need for a practical and reliable predictive tool to identify high-risk ECBM patients and provide personalized treatment strategies. In recent years, nomograms have emerged as intuitive and effective predictive tools, widely used for assessing the prognosis in malignancies. However, due to the rarity of ECBM, there are few related studies, and no comprehensive analyses have been published on the risk factors for early death in ECBM patients. Furthermore, there has been no research to establish predictive models for early mortality in ECBM. The nomogram based on SEER database has a larger total sample, which significantly improves the accuracy and stability of the nomogram. In this study, we explored the independent risk factors for early death in ECBM patients using the SEER database and developed easy-to-use nomogram models to predict the risk of early death. These models aimed at assisting clinicians in the early identification of high-risk patients and optimizing clinical decision-making.

The univariate and multivariate analyses identified five independent risk factors for OED in ECBM patients, including surgery, histological type, chemotherapy, brain metastasis, and time from diagnosis to treatment. Additionally, five independent risk factors were determined for CSED in ECBM patients, which include surgery, radiotherapy, chemotherapy, brain metastasis, and time from diagnosis to treatment. Among the independent risk factors for OED, chemotherapy had the most significant impact on patient prognosis, followed by time from diagnosis to treatment, surgery, histological type, and brain metastasis. Similarly, in the context of CSED, chemotherapy emerged as the most critical factor, followed by time from diagnosis to treatment, surgery, brain metastasis, and radiotherapy. Surgery is the standard treatment for localized EC. However, there remains controversy regarding the use of surgery for metastatic or advanced EC. Most ECBM patients experience varying degrees of pain and structural bone damage (10). The primary goals of surgery were alleviating symptoms (such as pain, fractures, and nerve compression), reducing tumor burden, improving function and quality of life, facilitating other treatments, and prolonging survival.

This study revealed that surgery was significantly associated with improved survival outcomes in ECBM patients, suggesting a potential protective effect, which is consistent with previous research (11). However, we acknowledge that in this retrospective analysis, the selection of surgical candidates may have been influenced by factors such as better baseline clinical condition, disease extent, and overall performance status. Currently, cytoreductive surgery has been demonstrated to enhance survival outcomes and prolong overall survival (OS) in appropriately selected ECBM patients (12, 13). In clinical practice, whether to perform surgery for ECBM patients should comprehensively consider the site of metastasis, degree of diffusion, respectability, and patient status. Similarly, this study found that chemotherapy was associated with survival benefits for ECBM patients, though we recognize that treatment selection may have been influenced by patient fitness and disease characteristics suitable for systemic therapy. The combination of carboplatin and paclitaxel has been established as a first-line treatment for advanced and metastatic EC patients (13). With ongoing research into TCGA molecular subtyping, the therapeutic landscape for metastatic and recurrent EC is changing. The National Comprehensive Cancer Network (NCCN) guidelines recommend the use of carboplatin and paclitaxel in combination with pembrolizumab or dostarlimab as first-line therapy for recurrent or metastatic EC patients (14). Two phase III clinical trials have shown that compared to chemotherapy alone, the use of immune checkpoint inhibitors (i.e., pembrolizumab or dostarlimab) in conjunction with conventional chemotherapy leads to improvements in both progression-free survival (PFS) and overall survival in patients with metastatic or recurrent EC patients (15), without a significant increase in the incidence of common adverse effects. This study indicates that radiotherapy is a weak influencing factor for CSED, which aligns with previous findings (11, 12). For patients with early moderate to high risk EC, vaginal brachytherapy (VBT) or external beam radiotherapy (EBRT) can effectively reduce tumor recurrence and mortality rates (16). However, some researchers argue that due to distant metastases in advanced EC patients, radiotherapy as a local treatment is difficult to effectively control distant metastases and improve the survival rate of advanced patients (17, 18). Therefore, for ECBM patients, palliative EBRT is commonly used for pain relief (19). To improve the survival of distant metastases patients, several recent studies have explored the performance of stereotactic ablative radiotherapy (SABRT) in patients with oligometastases, as shown in a Phase II randomized SABR-COMET trial (NCT01446744). SABRT significantly reduced the progression-free survival (PFS) in oligometastasis patients but prolonged OS (20). In order to improve the survival and prognosis of patients, the treatment of distant metastatic EC patients usually needs to be personalized and customized by evaluating the patient’s status, pathological classification, comprehensive surgery, radiotherapy, chemotherapy, targeted therapy and immunotherapy, etc.

Furthermore, this study found that histological type is also an important factor for ECBM patients, with sarcomatous subtypes being significantly associated with a higher risk of early death. According to the research, patients with sarcomatous subtypes often have poorer prognoses, particularly those with high-grade endometrial stromal sarcoma (9). Studies have shown that these subtypes are generally more aggressive and are often associated with high recurrence rates and low survival rates and that traditional treatments (including surgery, chemotherapy, and radiotherapy) offer limited survival benefits for these subtypes (21, 22). The relationship between multiple distant metastases and poor prognosis in EC patients has been confirmed (23). In this study, ECBM patients with concomitant brain metastases presented a higher risk of early death rate, which is consistent with previous studies (6, 23). Mao et al. revealed that the shortest median survival of two-organ metastasis with brain metastases was only 1 month, which may be related to the easy formation of multiple metastases in the brain and the difficulty of treatment due to the limitation of the blood-brain barrier the early mortality risk in ECBM patients (6, 24). Additionally, an extended time from diagnosis to treatment initiation could lead to disease progression and further worsen prognosis. This finding underscores the importance of early intervention, which is crucial for reducing the early death risk in ECBM patients.

This research revealed that age is not an independent predictor of early death for ECBM patients, which is consistent with previous studies (11, 17, 25). In this study, patients with bone metastases had an older median age, and shorter survival, which may be one of the important reasons why age was not included as an independent prognostic factor. Many previous studies have shown that tumor grade is an important factor influencing patient prognosis including metastatic EC (11, 17, 26), but it is not significant in this study, which may be due to the high proportion of missing data on pathological grade in this study, and future studies with higher data integrity are needed. In terms of pathological grade, young EC patients are often highly differentiated and staged early, which indicates a higher survival rate and better quality of life. Older women are usually diagnosed at an advanced stage with poorer histological types and tend to have a poor prognosis. Elderly patients are usually complicated with underlying diseases, are more likely to have risk factors such as invasion of tumor, advanced stage, and more aggressive histological type, and have poor tolerance to treatment. Therefore, age factors should be fully considered in clinical practice to optimize disease management strategies. Previous studies have found that tumor size is an important independent predictor of early EC patients (27). However, tumor size may not have a significant effect on the prognosis of metastasis patients, which was consistent with the findings of Yan et al. (28). Race was also not a significant independent predictor in this study, which is different from what previous studies found (29). This may be because many previous studies focused on all EC patients, but this study only focused on ECBM patients. Currently, few studies have found a link between survival and marital status.

Despite the inherent challenges associated with studying rare malignancies through retrospective database analysis, this research addresses a significant clinical need. The development of predictive nomograms for ECBM patients fills an important gap in gynecologic oncology, providing clinicians with evidence-based tools for risk stratification and treatment planning in a patient population that has been inadequately studied due to its low incidence. While the clinical scenario may represent a small proportion of gynecologic oncology practice, the high early mortality rate (41.5%) observed in ECBM patients emphasizes the critical importance of accurate prognostic assessment for optimal patient management. The rigorous statistical approach employed in this study, combined with the comprehensive nature of the SEER database, provides the most robust analysis possible within the constraints of studying this rare condition.

ROC curve, calibration curve, and DCA analysis of this research revealed that the nomograms have high prediction accuracy, high consistency, and clinical application value. However, this study has some limitations: (1) This study was established based on a public database and employed a retrospective design. Due to the low incidence of ECBM, external validation was not included in this study. Therefore, the models need to be further evaluated by external data from multiple institutions; (2) The SEER database does not provide the detailed records of chemotherapy, radiotherapy and targeted therapy, which limits the further exploration of treatment plans in this study; (3) The SEER database does not contain TCGA molecular typing (such as POLE mutation, MSI-H, etc.), tumor biomarkers (such as CA125, etc.), lymphatic vascular space infiltration (LVSI), immunohistochemistry, lymph node metastasis and other important information, which limits the comprehensiveness of the models. (4) The SEER database contains a large amount of unknown information, which may interfere with the results of the models. This study is a retrospective study, and the prediction accuracy of the model needs to be further verified by future multi-center prospective studies. (5) It is important to acknowledge that the clinical scenario evaluated in this study, ECBM, represents a relatively small proportion of gynecologic oncology practice. This relatively low prevalence may limit the broad applicability of our findings across the full spectrum of gynecologic oncology. Nevertheless, for this specific patient population, our nomograms provide valuable prognostic tools that can assist in clinical decision-making despite the limited overall incidence of the condition. While we acknowledge that this retrospective analysis based on the SEER database has inherent limitations, the rarity of this condition makes large-scale prospective studies challenging. Within the scope of available data, we employed rigorous statistical methodology to address clinically relevant questions and provide evidence-based prognostic tools where none previously existed.

## 5 Conclusion

The predictive models constructed in this study can effectively predict the risk of early death in ECBM patients, providing an important reference for clinical decision-making. Despite limitations, this study lays a foundation for improving the prognostic management of high-risk ECBM patients.

## Data Availability

The original contributions presented in the study are included in the article/**Supplementary Material**. Further inquiries can be directed to the corresponding author.
